# Exploring the Effects of Cerebellar tDCS on Brain Connectivity Using Resting‐State fMRI

**DOI:** 10.1002/brb3.70302

**Published:** 2025-02-09

**Authors:** Beatriz Catoira, Debora Lombardo, Stefanie De Smet, Raquel Guiomar, Peter Van Schuerbeek, Hubert Raeymaekers, Natacha Deroost, Frank Van Overwalle, Chris Baeken

**Affiliations:** ^1^ Department of Psychiatry (UZ Brussel) Vrije Universiteit Brussel Brussels Belgium; ^2^ Ghent Experimental Psychiatry (GHEP) Lab Ghent University Ghent Belgium; ^3^ DIBRIS University of Genova Genova Italy; ^4^ Brain Stimulation and Cognition (BSC) Lab, Department of Cognitive Neuroscience, Faculty of Psychology and Neuroscience Maastricht University Maastricht the Netherlands; ^5^ Center for Research in Neuropsychology and Cognitive and Behavioral Intervention, Faculty of Psychology and Educational Sciences University of Coimbra Coimbra Portugal; ^6^ Department of Radiology UZ Brussel Brussels Belgium; ^7^ Department of Psychology and Center for Neuroscience Vrije Universiteit Brussel Brussels Belgium; ^8^ Department of Electrical Engineering Eindhoven University of Technology Eindhoven the Netherlands

**Keywords:** cerebellum, default mode network, fMRI, mentalizing, tDCS

## Abstract

**Purpose:**

The cerebellum's role extends beyond motor control, impacting various cognitive functions. A growing body of evidence supports the idea that the cerebellum optimizes performance across cognitive domains, suggesting critical connectivity with the neocortex. This study investigates how cerebellar transcranial direct current stimulation (tDCS) targeting the right Crus II region modulates functional brain connectivity.

**Method:**

Using a within‐subject design, 21 healthy participants underwent both sham and anodal cerebellar tDCS at 2 mA during 20 min of concurrent resting‐state fMRI sessions. Data was preprocessed, and connectivity changes were examined using seed‐to‐voxel analysis. Given the potential impact of cerebellar dysfunctions on symptoms associated with autism spectrum disorders, we also assessed how individual autism quotient (AQ) scores might influence cerebellar functional connectivity. Moreover, electrical field simulations were computed for each participant to explore the effects of individual differences.

**Findings:**

Results indicated increased functional connectivity between the cerebellar Crus II and the right inferior frontal gyrus (IFG) during active tDCS compared to sham stimulation. The IFG (part of the Action Observation Network) plays a crucial role in understanding the actions and intentions of others, implicating the cerebellum in higher‐order cognitive processes. In addition, linear mixed‐effects models revealed an interaction between electric field strength and AQ scores, suggesting that functional connectivity changes are based on individual psychobiological differences.

**Conclusion:**

Cerebellar tDCS significantly altered functional brain connectivity, particularly between the cerebellar Crus II and the IFG, both involved in social cognition. These findings contribute to our understanding of the cerebellum's role beyond motor control, highlighting its impact on cognitive and social processes and its potential for therapeutic applications, such as autism spectrum disorders.

## Graphical abstract

Cerebellar tDCS during the resting state increased functional connectivity between the cerebellar Crus II and the right inferior frontal gyrus during active tDCS compared to sham stimulation. In addition, we found an interaction between electric field strength and autism quotient scores, suggesting functional connectivity changes based on individual differences.

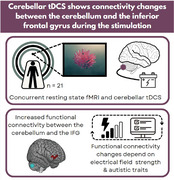

## 1 Introduction

The cerebellum's involvement in various cognitive functions beyond motor control suggests a complex network of connectivity with the rest of the brain. The function of the cerebellum has been hypothesized to be the same across multiple domains (such as language or social cognition) as proposed by the Universal Cerebellar Transform (UCT) theory (Schmahmann 1996). The UCT theory originated after an in‐depth exploration of the anatomical connections between the cerebellum and the associative and paralimbic cortex, which suggested that the cerebellum performs the same computations for these cortices as it does for the sensorimotor system (Schmahmann 1996). According to this theory, in the same way, the cerebellum performs coordination and fine‐tuning of motor actions (such as modulating how much we need to tilt a glass of water to drink from it), the cerebellum also coordinates and fine‐tunes other complex cognitive processes such as understanding when to intervene in a group conversation.

More recent findings in mouse models support the idea that cerebellar involvement in multiple domains might be mediated by the anatomical projections of different cerebellar areas (Gornati et al. 2018). Specifically, it has been hypothesized that regions of the cerebellum that send projections through the thalamic laminar nuclei can modulate the interactions of multiple brain regions, whereas the regions that project through the ventral lateral nucleus and the ventral posterolateral nucleus in the thalamus may transmit information to more focal neocortical areas (Gornati et al. 2018). Furthermore, recent research suggests that these connections form open loops (Biswas et al. 2019) that become increasingly functionally integrated after repeated cerebellar‐neocortical pairings (Wagner et al. 2019). In line with previous findings, the cerebellar area Crus II has been shown to send connections to the dorsolateral prefrontal cortex in monkeys (Kelly and Strick 2003). In humans, lesions of the left Crus I, lobule VI, and disruptions in the cerebello‐cortical pathways, particularly through the left frontostriatal tract and the left thalamic projection, are more likely to contribute to deficits in social cognition (Beuriat et al. 2022). Also in humans, a large set of studies has found cerebellar activation in social mentalizing, particularly in tasks that require sequential coordination of social actions and predictions (for a review see Van Overwalle 2024).

Following the idea that the cerebellum is involved in multiple cognitive domains, there have been multiple attempts at parcellating the cerebellum according to its connectivity with the cortex during resting state (Buckner et al. 2011; Metoki, Wang, and Olson 2022) and task‐based (King et al. 2019) functional magnetic resonance imaging (fMRI). Both of these approaches have led to the conclusion that the bilateral Crus II of the cerebellum is mostly correlated to cortical areas of the default mode network (DMN) at rest (Buckner et al. 2011; Metoki, Wang, and Olson 2022) and using a battery of 26 tasks (King et al. 2019). A more recent task‐related meta‐analysis based on the NeuroSynth database also confirmed the link between Crus II and the mentalizing network (Van Overwalle et al. 2024), which is a major component of the DMN (Schurz et al. 2021).

Further evidence of the involvement of the cerebellum with mentalizing comes from the study of pathological conditions. The cerebellar insult at birth (Limperopoulos et al. 2007), undergrowth (Sparks et al. 2002), and lesions in adulthood (Koziol et al. 2014) result in an increase in autism diagnosis or autism‐like social deficits. In line with these findings, reduced gray matter volume in Crus I and Crus II (D'Mello et al. 2015; Yaxu et al. 2020) and functional connectivity between Crus I and the posterior superior temporal gyrus (Jack and Morris 2014) have been correlated with the severity of autistic symptoms measured by questionnaires such as the autism quotient (AQ; Baron‐Cohen et al. 2001). Moreover, structural and functional differences in the cerebellum have been extensively reported in autism spectrum disorders (for a review see Crippa et al. 2016). Altered connectivity between the cerebellum and the DMN has also been observed across several clinical conditions (Phillips et al. 2015), such as schizophrenia (Shinn et al. 2015), depression (Liu et al. 2012; Wang et al. 2023), or posttraumatic stress disorder (Holmes et al. 2018). In addition, patients with a *SCA2* mutation have shown altered resting state connectivity between the right Crus II and mentalizing regions such as the dorsomedial prefrontal cortex and the temporoparietal junction (Olivito et al. 2024).

An emerging promising tool to treat psychiatric disorders is transcranial direct current stimulation (tDCS), a non‐invasive brain stimulation method that can be used to increase or inhibit specific brain areas. Cerebellar tDCS has proven to be effective at targeting specifically the social functions of the cerebellum (Catoira et al. 2023; Oldrati et al. 2021; Rice, D'Mello, and Stoodley 2021). However, its effects remain unclear (Oldrati and Schutter 2018; Pezzetta et al. 2024), with some studies finding anodal tDCS to have an inhibitory effect (Ballard et al. 2019) and others finding a facilitatory effect (Ferrucci et al. 2012, Ferrucci et al. 2013) in learning, social, or mentalizing tasks. Along with the increase in popularity of the cerebellum as a target for tDCS, the field of computational tDCS simulations has begun to explore how different montages and variability between individuals affect the electric field generated by tDCS (Gomez‐Tames et al. 2019; Klaus and Schutter 2021).

In an attempt to make a closer examination of the effects of cerebellar tDCS, some studies have paired tDCS with neuroimaging techniques, such as fMRI (Catoira et al. 2023; Haihambo et al. 2024; Rice, D'Mello, and Stoodley 2021; Stoodley et al. 2017). By combining tDCS with fMRI, we can not only measure behavioral effects, but we can also take a closer look at changes in brain activity and connectivity. Although these studies have explored the effects of cerebellar tDCS on brain activity during mentalizing tasks (Catoira et al. 2023; Haihambo et al. 2024; Rice, D'Mello, and Stoodley 2021; Stoodley et al. 2017), the focus has been on effects after stimulation has ended, whereas little is known about the effects of cerebellar tDCS versus sham during the actual period of stimulation. Since connectivity patterns during tDCS may differ from those observed afterward, concurrent connectivity measurements can reveal how tDCS modulates networks immediately, laying the groundwork for understanding its role in short‐ and long‐term plasticity and behavioral changes. Also, while task‐based paradigms show process‐specific modulation, resting‐state measurements establish baseline effects, free from task‐related variability, and provide insights into network‐wide connectivity changes unconstrained by task‐specific activation.

In this concurrent tDCS‐resting‐state fMRI study, we primarily investigated how anodal tDCS targeting the right Crus II modulates functional brain connectivity, using the right Crus II as the seed region of interest. Our main research question focused on identifying connectivity changes induced by cerebellar stimulation. Secondarily, we explored whether these connectivity changes were influenced by individual differences, such as the intensity of the electrical field (EF) experienced by each participant. Furthermore, given the established link between the cerebellum and autistic traits, we conducted an exploratory analysis to assess whether AQ scores might moderate the observed effects, aiming to provide preliminary insights into the role of individual characteristics in shaping tDCS outcomes.

## 2 Methods

### 2.1 Participants

For this study, 23 healthy right‐handed participants between 18 and 35 years old were recruited using flyers posted on social media. Due to issues with poor brain imaging quality, only 21 participants were analyzed (*x̄ *= 25 years, SD = 3.99, 5 males and 16 females). All participants were screened for MRI and tDCS safety. Screening also included the modified version of the MINI screen (Mini International Neuropsychiatric Interview, version 5.0.0; Lecrubier et al. 1998; Dutch version by Overbeek et al. 1999), the Beck Depression Inventory (BDI‐II; Beck et al. 1996; Dutch version by van der Does 2002) and the AQ (Baron‐Cohen et al. 2001; Dutch version by Hoekstra et al. 2008) questionnaires. Volunteers with current psychiatric disorders or medication, clinical scores of depression or autism (> 14 on the BDI or > 32 in the AQ), were excluded from participation.

### 2.2 Procedure

This study used a within‐subject design in which participants came for two sessions (active session and sham session) in a counterbalanced order. The time between the sessions ranged from 1 day to 14 days (*x̄ *= 4.7 days).

Each session started with signing the informed consent and an explanation of the procedures, followed by the positioning of the tDCS electrodes. Afterwards the participant was placed inside the scanner. While in the scanner, each participant underwent anatomical scans, 7 min of resting state pre‐stimulation (while only looking at a fixation cross), 20 min of resting state with sham/stimulation (including the projection of a movie designed for resting state), 7 min of resting state poststimulation (with just a fixation cross), and 15 min of a pictorial sequencing task.

Upon completion of the two sessions of the study, participants received monetary compensation. The study was approved by the Medical Ethics Committee of the Vrije Universiteit Brussel (Universitair Ziekenhuis Brussel).

### 2.3 Resting State During Stimulation

To minimize head movement during the 20 min of resting state with stimulation and to increase test–retest reliability, the movie Inscapes (Vanderwal et al. 2015) was shown. Inscapes is a 7‐min computer‐generated animation that consists of colored moving shapes; the content is nonverbal, nonsocial, and has no narrative arc. The movie was looped three times to account for the total duration of the stimulation.

### 2.4 tDCS Protocol

tDCS was administered using two rubber electrodes, with dimensions of either 4.5 × 4.5 cm or 5 × 5 cm (the variation in electrode size was due to technical issues and the subsequent unavailability of a single size). The tDCS device used for stimulation was the MRI‐compatible model 1300A from Soterix Medical (New York, NY, USA). The electrodes were affixed to the participants' skin using Ten20 Paste gel and secured to the head with rubber bands.

**FIGURE 1:**
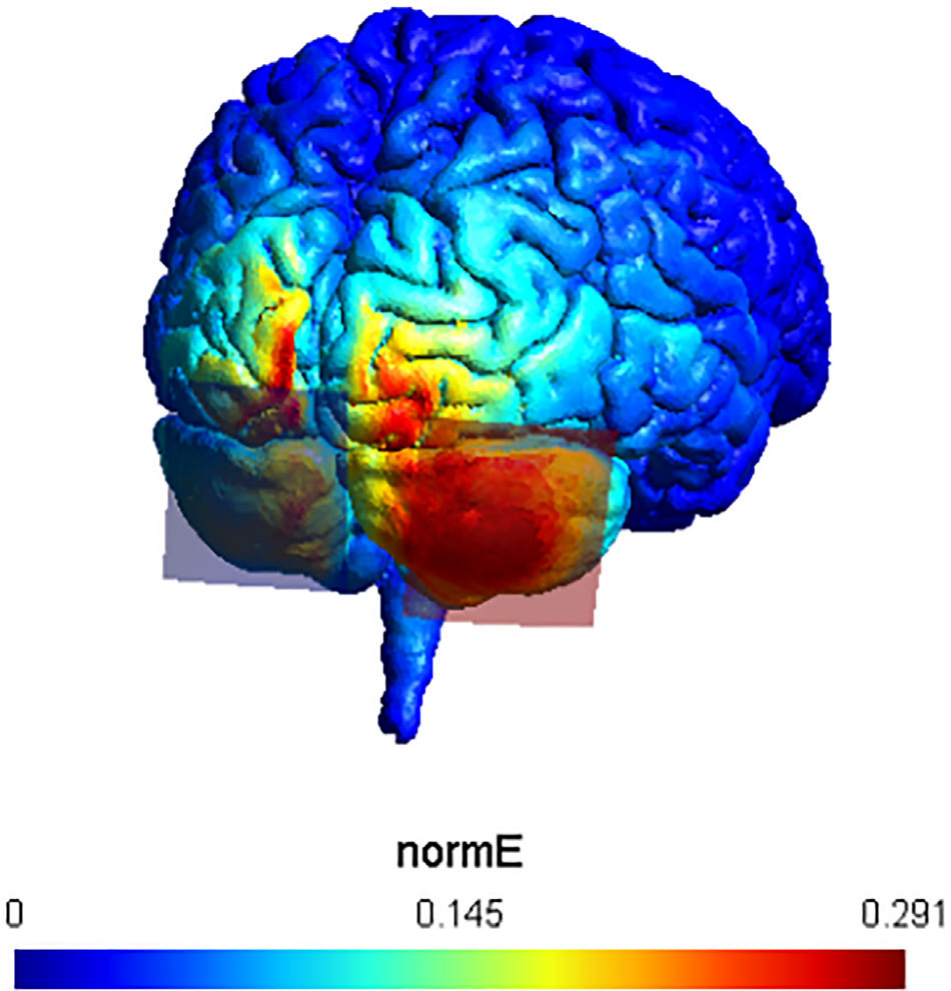
tDCS montage on the head of Ernie provided by SimNIBS. The anode (semitransparent in red, on the right) is located over PO10, and the cathode (semitransparent in blue, on the left) is located over Iz to maximize focality and intensity of the current on the Crus II of the cerebellum.

To accurately target the right posterior cerebellum at MNI coordinates (*X* = 25, *Y* = −75, *Z* = −40), multiple simulations were conducted on a model head using SimNibs (version 3.2.1; Thielscher et al. 2015). The objective was to identify the electrode configuration that would optimize the focality of anodal stimulation in this area. The optimal setup was found to have the anode placed over the PO10 position of the extended 10–20 EEG system and the cathode over Iz (for a visualization of the montage see Figure 1). The Iz electrode was located following anatomical landmarks, and PO10 was positioned by measuring 10% of the distance between Iz and Nz, crossing through A10. The positioning was later confirmed upon visual inspection of the SimNibs simulations.

For anodal stimulation, the current ramped up over 30 s initially, maintained an intensity of 2 mA for 20 min and then ramped down over the final 30 s. In the sham condition, we used the sham built‐in function from Soterix, which mimicked the anodal setup by 30 s ramping up and down at the beginning and end of the 20 min period, but no current was applied during the interim period.

### 2.5 fMRI Acquisition

FMRI data was acquired in a 3T Discovery MR750w with a 24‐channel head coil (GE Medical Systems, Milwaukee, WI, USA) at the UZ Brussel in Brussels, Belgium. First, we obtained a T1‐weighted anatomical scan using a 3D BRAVO sequence (432 sagittal slices, 0.8 mm slice thickness, FoV 25.6 × 25.6 mm, TR = 8.2 ms, TE = 3.2 ms, TI = 450 ms, flip angle = 12°). During the resting state, gradient multi‐echo functional scans were acquired (57 slices per location, slice thickness of 2.5 mm, FoV 22.0 × 22.0 mm, 2 echoes: TE1 = 22.5, TE2 = 62.6 ms, TR = 2000 ms, flip angle = 52°). The acquisition type was continuous.

### 2.6 fMRI Preprocessing and Analysis

The initial step involved converting the raw images acquired in DICOM format into NIFTI files using MRIcroGL (Rorden and Brett 2000). For preprocessing the images, SPM 12 was utilized (Wellcome Department of Cognitive Neurology, London, UK).

All images were reoriented to the AC‐PC plane, and a 4D‐3D file conversion was performed. To merge the multi‐echo images, we used the SPM‐based Multi Echo and Hyperband toolbox (MEHB fMRI; https://github.com/P‐VS/MEHBfMRI). Next, to eliminate susceptibility artifacts, the ACID toolbox (http://diffusiontools.com/) was employed, which is based on a phase polarity inverted scan method. Subsequently, slice time correction was performed using the MEHB toolbox. Functional images were then coregistered to the anatomical T1 image, normalized to the MNI‐152 template, and smoothed with a Gaussian kernel of 8 mm FWHM. Finally, data were examined for excessive motion using the Artifact Detection Tool (ART; http://web.mit.edu/swg/art/art.pdf; http://www.nitrc.org/projects/artifact_detect); however, none of the included participants had to be removed due to excessive motion.

To remove additional confounding effects due to motion, physiological sources, or other artifactual effects, denoising was applied. This step was conducted using CONN (v.22.v.2407; Whitfield‐Gabrieli and Nieto‐Castanon 2012). Motion was defined as a first‐level covariate containing six time series, each representing the estimated participant movement with translation and rotation parameters. Gray matter, white matter, CSF, and motion were included as confounding effects. The filter was applied in the range of [0.008–0.09] Hz, with linear detrending.

Connectivity analyses were performed using the CONN toolbox. The first‐level connectivity measure was computed using Pearson's correlation between the Crus II BOLD time series and each individual voxel BOLD time series in the rest of the brain from a weighted general linear model (weighted‐GLM). The ROI was defined as a sphere with a 10 mm radius, centered at the MNI coordinates (*X* = 25, *Y* = −75, *Z* = −40, based on meta‐analysis (Van Overwalle et al. 2014; Van Overwalle, Ma, and Heleven 2020). To stabilize the variance and make the data normally distributed, the correlation coefficients were converted to *z*‐maps using Fisher's transform. For the group‐level analysis, a population‐level inference was performed from the resulting connectivity measures of each first‐level analysis. For each individual voxel, a separate GLM was estimated, with first‐level connectivity measures at this voxel as dependent variables (one independent sample per participant and one measurement per experimental condition).

Voxel‐level hypotheses were evaluated using multivariate parametric statistics with random effects across participants and sample covariance estimation across multiple measurements. Inferences were performed at the level of individual clusters (groups of contiguous voxels). Cluster‐level inferences were based on parametric statistics from Gaussian random field theory. Functional connectivity strength was represented by Fisher‐transformed bivariate correlation coefficients from a weighted GLM. For the second level of analysis, we added as covariates age (mean centered), gender, and stimulation order (whether stimulation was applied on the first or the second session).

For this study, we performed a seed‐to‐voxel analysis, taking into account the connectivity between the right Crus II and the rest of the brain and the differences between sham and active cerebellar tDCS (stimulation > sham) during the 20 min block of stimulation. Differential functional connectivity (between‐group analysis) was performed by weighing the connectivity with a [1–1] contrast. Results were thresholded using a combination of a cluster‐forming *p* < 0.005 voxel‐level threshold and a familywise corrected *p*(FDR) < 0.05 cluster‐size threshold.

### 2.7 tDCS Simulations

To compute the intensity of the EF generated by the stimulation on each participant, we used SimNIBS. We first segmented the T1 image of the stimulation session of each participant. After that we simulated the tDCS montage specified above. Then the current intensity in the same cerebellar ROI with a 10 mm radius used for the seed of the connectivity analysis was extracted.

### 2.8 Linear Mixed‐Effects Models

To evaluate the relationship between connectivity and the strength of the EF, we computed linear mixed‐effects models using R 4.2.3 and RStudio/2022.12.0 + 353 (lmerTest package). We used the functional connectivity measures extracted from CONN (between the seed and the significant cluster) as the dependent variable. To account for inter‐participant variability, we included subject as a random effect. For the fixed effects, we included stimulation (sham or active), EF, and AQ scores as fixed predictors. EF and AQ were scaled (using the *scale* function of R, which computes a *z*‐score), and we followed the formula: “FC ∼ roi_EFscaled*AQscaled + Stimulation + (1+|participant).” Different models (Gamma with identity link, Gamma with a log link, inverse Gaussian with a log link, and Gaussian with a log link) were tested, and the best fit was chosen according to the Akaike Information Criterion.

## 3 Results

### 3.1 Functional Connectivity

**FIGURE 2:**
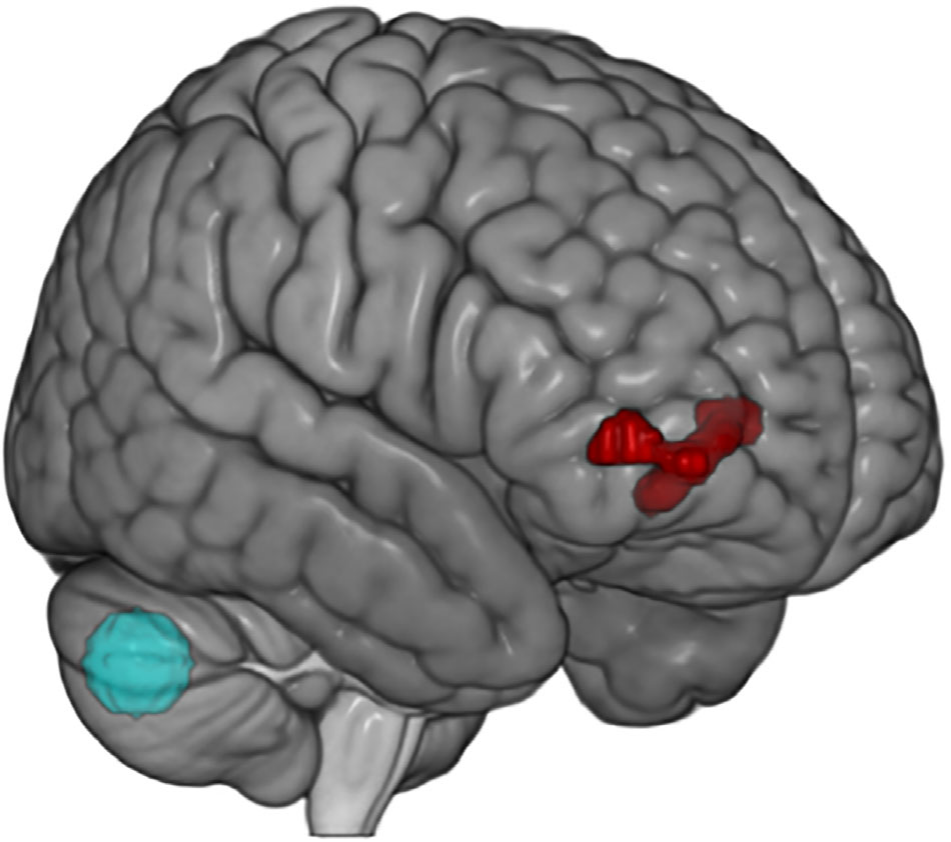
Right inferior frontal gyrus cluster. This cluster (in red) presented a significant increase in connectivity with the cerebellar seed (right Crus II, in light blue) during active stimulation compared to sham.

The seed‐to‐voxel analysis revealed a significantly stronger connectivity pattern during active as compared to sham stimulation between the right Crus II and the right inferior frontal gyrus (IFG; see Table 1). The cluster spread across the superior frontal gyrus, and inferior orbitofrontal cortex, with the peak located in the right IFG (see Figure 2).

**TABLE 1:** Results of the seed‐to‐voxel connectivity of right Crus II when contrasting active > sham stimulation.

Anatomical label	Cluster‐level				Peak‐level		Peak MNI coordinates		
Right inferior frontal gyrus	size	*p*(FWE)	*p*(FDR)	*p*‐unc	*p*(FWE)	*p*‐unc	*x*	*y*	*z*
	345	0.019	0.017	0.00028	0.552	0.000008	+50	+46	−04

### 3.2 Linear Mixed‐Effects Models

**FIGURE 3:**
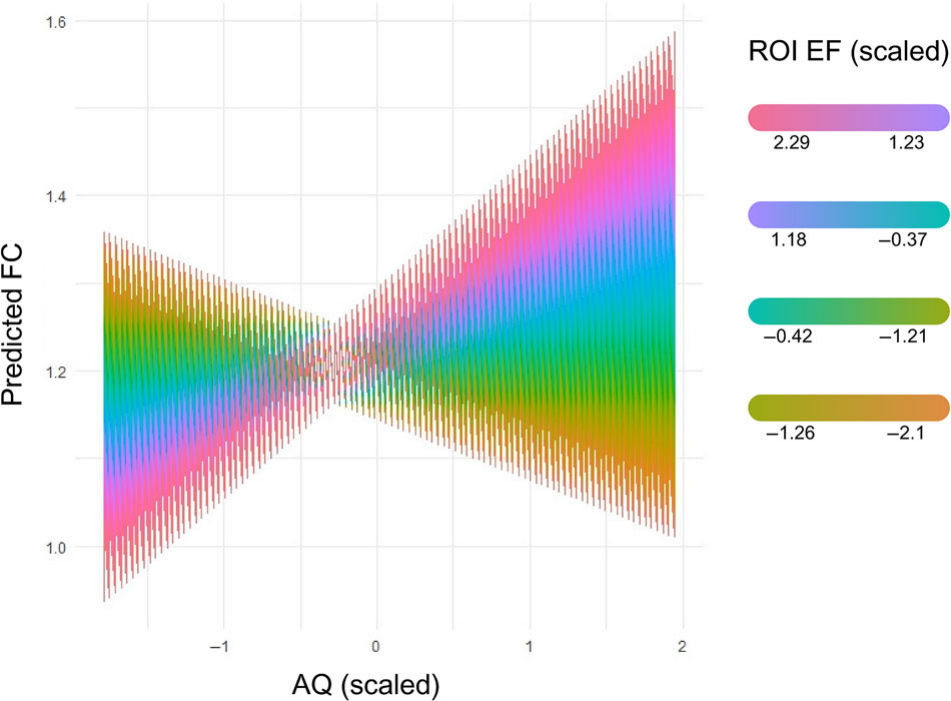
Interaction between autism quotient (AQ) scores and electrical field intensity (EF) in the Crus II ROI as explanatory factors in the changes in functional connectivity (FC) when comparing stimulation to sham. AQ on the *X*‐axis has been scaled. EF on the cerebellar ROI has also been scaled, and it is displayed on a gradient that goes from orange (low EF) to pink (high EF). In this figure we can observe how, for example, for participants with low AQ scores (to the left of the *X*‐axis), a smaller EF (in orange) results in a higher increase in FC (on the *Y*‐axis), while for participants with a higher EF (in pink), there is less change in FC when comparing stimulation to sham. For participants with a high AQ (on the right side of the *X*‐axis), the opposite is also true: for participants with a low EF (orange), there is less increase in connectivity when compared to participants with a high EF (in pink).

The dependent variable for this analysis was the change in functional connectivity when comparing active and sham between the cerebellar seed and the IGF cluster. The analysis revealed a significant main effect of stimulation, *χ* ^2^(1, *N* = 21) = 65.489, *p* < 0.001. Although the main effects of AQ and EF were not significant, we did find a significant interaction between both factors, *χ* ^2^(1, *N* = 21) = 4.444, *p* < 0.05 (FDR corrected). As we can see in Figure 3, the impact of the EF on the change in functional connectivity between Crus II and the IFG is modulated by the AQ score. For those participants with higher AQ scores, FC increases with the intensity of the EF; in contrast, for those with lower AQ scores, their FC was the strongest with lower currents (lower EF).

## 4 Discussion

In this study, we used concurrent tDCS‐resting state fMRI to explore whole‐brain connectivity differences during cerebellar tDCS compared to sham in healthy participants. By targeting the right Crus II, we observed an increase in connectivity between the cerebellum and the right IFG. Although we did not confirm our hypothesis that the differences in the changes in functional connectivity could be explained by the intensity of the EF, we found an interaction between the intensity of the electric field and the AQ scores. Specifically, for individuals with lower AQ scores, increased current intensity resulted in fewer changes in the cerebellar‐right IFG functional connectivity, while for those with higher AQ scores, increased current intensity led to the opposite effect, increasing connectivity.

Although tDCS is becoming an emerging research and clinical tool in the field of social cognition, a recent meta‐analysis (Pezzetta et al. 2024) has emphasized the lack of consistency in the effects of lateralized anodal tDCS. Moreover, findings from motor studies have highlighted the impact of individual characteristics on the effects of tDCS (Laakso et al. 2015). Individual characteristics, such as the thickness of the skull, have an influence on the amount of electricity that effectively reaches the brain (Laakso et al. 2015), which is a determinant factor because it has also been proven that the effects of tDCS are dependent on the intensity used (Oldrati and Schutter 2018; Pezzetta et al. 2024). Specifically, the spatial resolution of tDCS could be limited by the skin‐cerebellum distance, resulting in significant variability in the efficacy of tDCS to modulate the cerebellum (Oldrati and Schutter 2018).

In this study we confirmed that the effects of cerebellar tDCS were dependent on individual characteristics, which, in addition to previous findings, highlights the need to take into account individual characteristics with the goal of obtaining a better understanding of when cerebellar tDCS is effective. Nonetheless, most aims at personalization of tDCS protocols require a previous MRI of each participant, which might not always be affordable; therefore, the potential use of questionnaires in combination with other techniques, such as measuring head circumference (Antonenko et al. 2021), to adjust the intensity of stimulation might be a promising line of research.

The cerebellum has been consistently identified as a key region within the mentalizing network (Haihambo et al. 2024; Heleven, van Dun, and Van Overwalle 2019; Van Overwalle et al. 2024; Van Overwalle et al. 2020). Specifically, dynamic causal modeling studies have shown that the cerebellum has ipsilateral (as well as contralateral) connections to cerebral areas of the mentalizing network (Van Overwalle et al. 2020). Previous research from our lab (Catoira et al. 2023) found that cerebellar tDCS modulates the mentalizing network, particularly affecting the TPJ and precuneus during mentalizing tasks. Although in this study we did not find a modulation of those areas, recent research emphasizes the IFG's involvement in social processes, particularly cognitive and affective functions. A novel meta‐analysis showed that the bilateral IFG is involved in both cognitive and affective aspects of social processing, with the right IFG being specifically associated with affective tasks (Schurz et al. 2021). In addition, the right IFG is a crucial component of the Action Observation Network (Rizzolatti and Craighero 2004), which activates during action observation. Along with the IFG, when observing actions that require more thought (such as when an action is unexpected or fails on its goal), regions of the mentalizing network are also recruited (Spunt and Lieberman 2013). Taken together, these findings suggest that the cerebellum might be functionally connected to the IFG via the mentalizing network.

Furthermore, studies on autistic participants, who often experience challenges with mentalizing tasks, have shown reduced connectivity between the bilateral IFG and other mentalizing regions, such as the TPJ, during tasks that involve observing emotional faces and hand actions (Jayashankar et al. 2023). Some studies have used multiple sessions of prefrontal (Ratsapbhayakul et al. 2024) and cerebellar (Hadoush and Hadoush 2023) tDCS and have found positive effects regarding autism symptoms and increased brain activation in mentalizing regions. Although in this study we did not include participants with scores > 32 in the AQ, and therefore we cannot generalize to the clinical autistic population, the interaction observed between EF and autistic traits (AQ) might have implications for studies aiming at personalization of tDCS protocols. The fact that participants with higher AQ scores may respond stronger to higher‐intensity electric fields, while those with lower AQ scores may be more sensitive to lower intensities, suggests that different participants may require different levels of stimulation for optimal results. Nonetheless, these findings must be interpreted with caution due to our modest sample and lack of clinical autism scores.

One of the main strengths of this study is the use of prolonged resting‐state analysis. While many studies rely on shorter runs to evaluate functional connectivity, we compared 20 min of active tDCS with 20 min of sham stimulation, providing a more extended measure of connectivity changes. In addition, we utilized the movie *Inscapes* during fMRI to minimize head movement and enhance the homogeneity of resting‐state data, a method previously validated in autistic children (Gabrielsen et al. 2018). Another strength is our use of individualized electric field modeling, accounting for anatomical variability and optimizing stimulation over the cerebellum. The within‐subjects design also adds robustness by controlling for individual variability in brain connectivity.

However, the relatively small sample size can be considered a limitation, as it may increase heterogeneity in both the spread of the stimulation current and the brain's connectivity patterns. To mitigate this, we employed a within‐subjects design and the *Inscapes* movie to reduce test‐retest variability, as previous studies have demonstrated its effectiveness in stabilizing connectivity measures (Vanderwal et al. 2015). Another potential concern is the possibility of false positives due to tDCS‐induced artifacts in the fMRI signal (Antal et al. 2014; Nardo et al. 2021). However, these artifacts tend to be localized at the stimulation site and are unlikely to influence the remote effects we observed. Moreover, future resting state fMRI tDCS studies should consider conducting resting state scans after stimulation to determine if changes in Crus II functional connectivity from active tDCS persist beyond the stimulation period.

## 5 Conclusion

In conclusion, our findings show that targeting the right Crus II with anodal tDCS increases connectivity between the cerebellum and the right IFG, a key component of the Action Observation Network. These changes in connectivity were linked to individual scores of EFs and autistic traits. This result is consistent with broader findings linking the cerebellum to the mentalizing network, where it plays a crucial role in social cognition. To conclude, the cerebellum could be a valuable target for non‐invasive brain stimulation to enhance social and cognitive functions. While our results are exploratory and should be interpreted with caution, they offer promising avenues for using tDCS to modulate mentalizing abilities in individuals with autism.

## Author Contributions

**Beatriz Catoira**: conceptualization, data curation, formal analysis, investigation, methodology, visualization, writing–original draft, writing–review and editing. **Debora Lombardo**: formal analysis, writing–original draft. **Stefanie De Smet**: formal analysis, writing–review and editing. **Raquel Guiomar**: formal analysis, writing–review and editing. **Peter Van Schuerbeek**: data curation, methodology, writing–review and editing. **Hubert Raeymaekers**: methodology, writing–review and editing. **Natacha Deroost**: funding acquisition, writing–review and editing. **Frank Van Overwalle**: conceptualization, funding acquisition, project administration, supervision, writing–review and editing. **Chris Baeken**: conceptualization, formal analysis, funding acquisition, project administration, resources, supervision, writing–original draft, writing–review and editing.

## Conflicts of Interest

The authors declare no conflicts of interest.

### Peer Review

The peer review history for this article is available at https://publons.com/publon/10.1002/brb3.70302.

## Data Availability

The data that support the findings of this study are available from the corresponding author upon reasonable request.
